# Supplementary material for: Adaptive Proteomic Changes in Protein Metabolism and Mitochondrial Alterations Associated with Resistance to Trastuzumab and Pertuzumab Therapy in HER2-Positive Breast Cancer
Source: Int J Mol Sci. 2025 Feb 12;26(4):1559. doi: 10.3390/ijms26041559 (PMC11855744; doi:10.3390/ijms26041559)
Supplement: Supplementary file 1 [file ijms-26-01559-s001.zip › ijms-3403005-supplementary.pptx]

## Slide 1
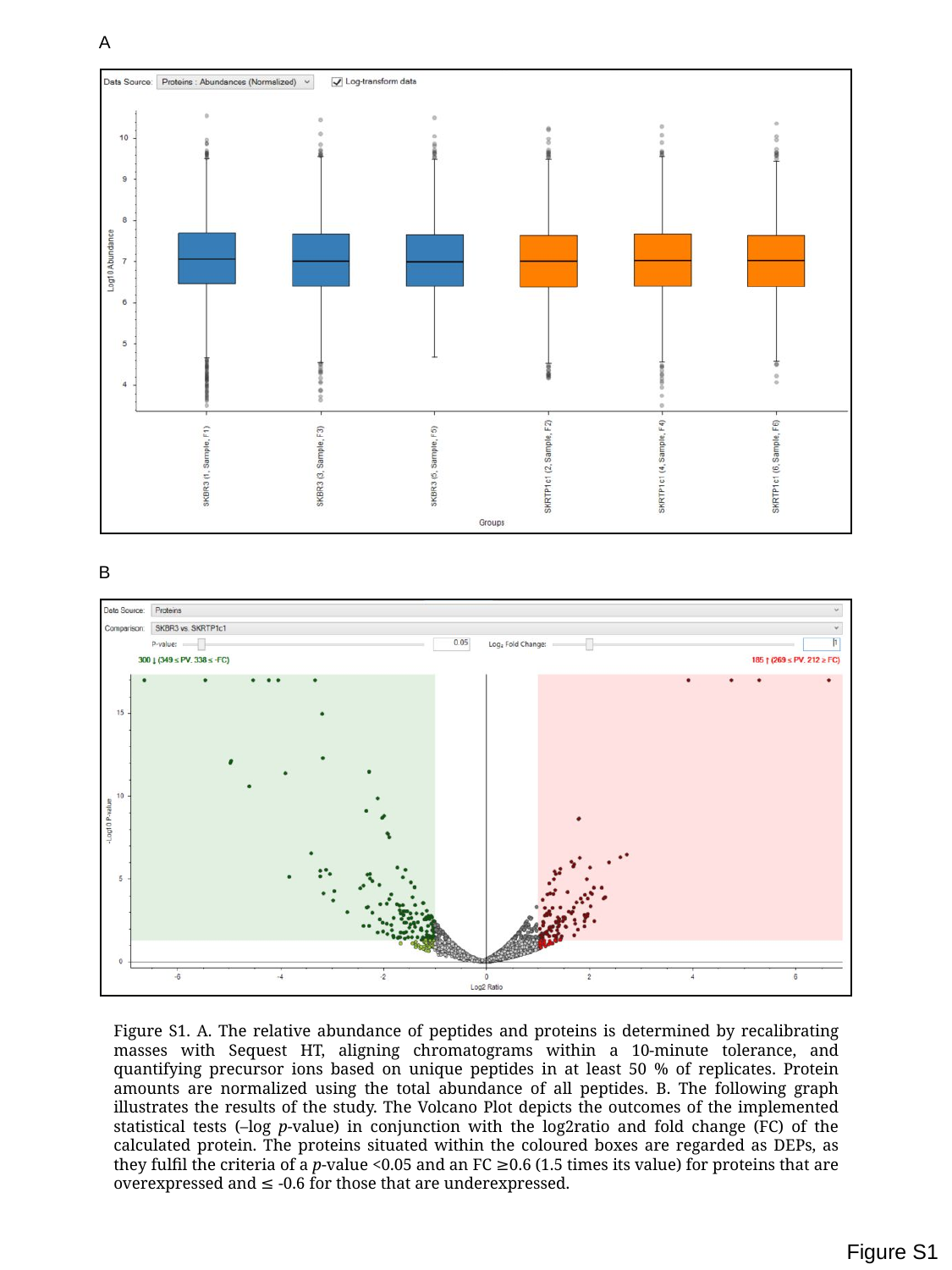

A
B
Figure S1. A. The relative abundance of peptides and proteins is determined by recalibrating masses with Sequest HT, aligning chromatograms within a 10-minute tolerance, and quantifying precursor ions based on unique peptides in at least 50 % of replicates. Protein amounts are normalized using the total abundance of all peptides. B. The following graph illustrates the results of the study. The Volcano Plot depicts the outcomes of the implemented statistical tests (–log p-value) in conjunction with the log2ratio and fold change (FC) of the calculated protein. The proteins situated within the coloured boxes are regarded as DEPs, as they fulfil the criteria of a p-value <0.05 and an FC ≥0.6 (1.5 times its value) for proteins that are overexpressed and ≤ -0.6 for those that are underexpressed.
Figure S1

## Slide 2
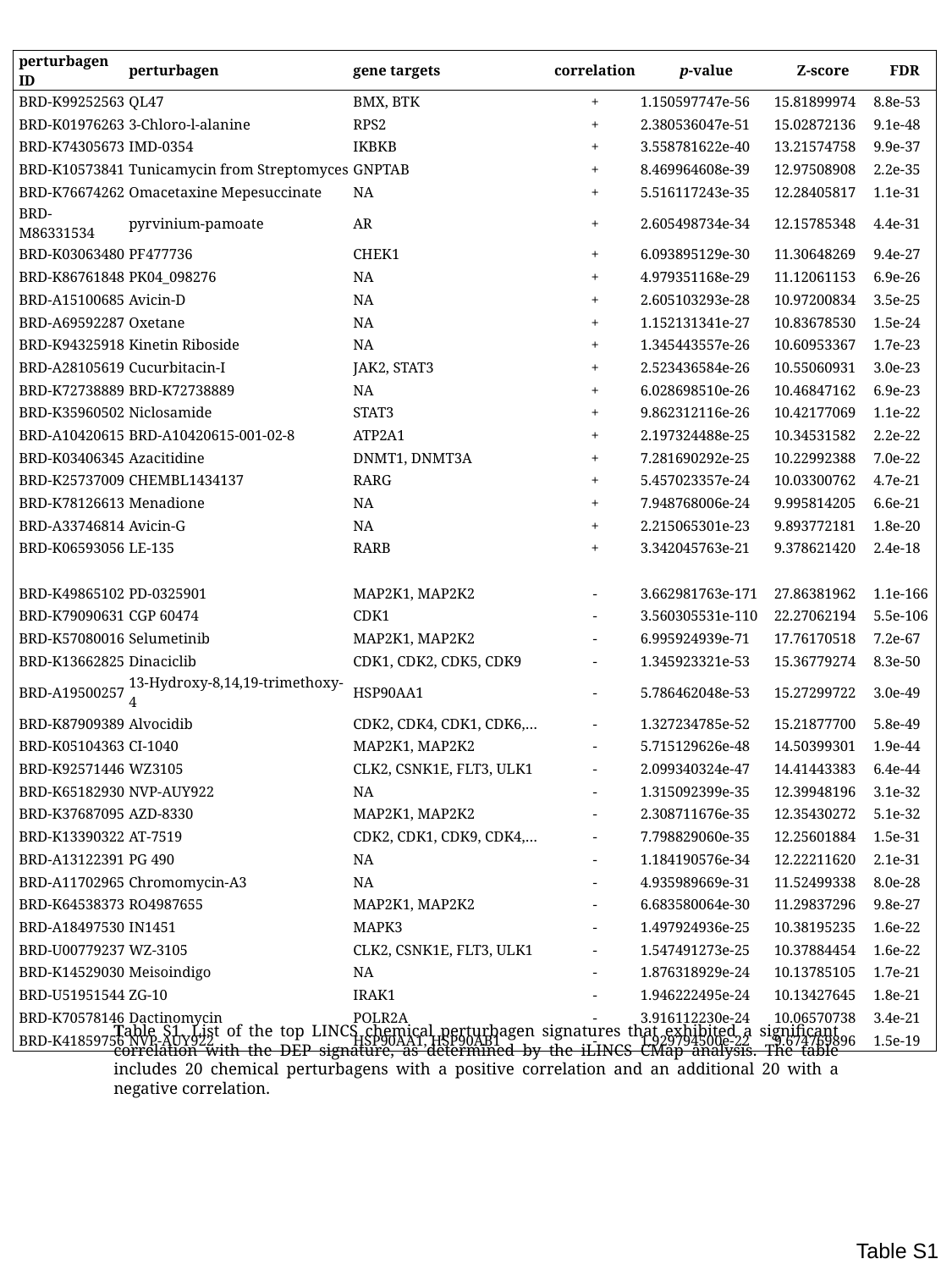

| perturbagen ID | perturbagen | gene targets | correlation | p-value | Z-score | FDR |
| --- | --- | --- | --- | --- | --- | --- |
| BRD-K99252563 | QL47 | BMX, BTK | + | 1.150597747e-56 | 15.81899974 | 8.8e-53 |
| BRD-K01976263 | 3-Chloro-l-alanine | RPS2 | + | 2.380536047e-51 | 15.02872136 | 9.1e-48 |
| BRD-K74305673 | IMD-0354 | IKBKB | + | 3.558781622e-40 | 13.21574758 | 9.9e-37 |
| BRD-K10573841 | Tunicamycin from Streptomyces | GNPTAB | + | 8.469964608e-39 | 12.97508908 | 2.2e-35 |
| BRD-K76674262 | Omacetaxine Mepesuccinate | NA | + | 5.516117243e-35 | 12.28405817 | 1.1e-31 |
| BRD-M86331534 | pyrvinium-pamoate | AR | + | 2.605498734e-34 | 12.15785348 | 4.4e-31 |
| BRD-K03063480 | PF477736 | CHEK1 | + | 6.093895129e-30 | 11.30648269 | 9.4e-27 |
| BRD-K86761848 | PK04\_098276 | NA | + | 4.979351168e-29 | 11.12061153 | 6.9e-26 |
| BRD-A15100685 | Avicin-D | NA | + | 2.605103293e-28 | 10.97200834 | 3.5e-25 |
| BRD-A69592287 | Oxetane | NA | + | 1.152131341e-27 | 10.83678530 | 1.5e-24 |
| BRD-K94325918 | Kinetin Riboside | NA | + | 1.345443557e-26 | 10.60953367 | 1.7e-23 |
| BRD-A28105619 | Cucurbitacin-I | JAK2, STAT3 | + | 2.523436584e-26 | 10.55060931 | 3.0e-23 |
| BRD-K72738889 | BRD-K72738889 | NA | + | 6.028698510e-26 | 10.46847162 | 6.9e-23 |
| BRD-K35960502 | Niclosamide | STAT3 | + | 9.862312116e-26 | 10.42177069 | 1.1e-22 |
| BRD-A10420615 | BRD-A10420615-001-02-8 | ATP2A1 | + | 2.197324488e-25 | 10.34531582 | 2.2e-22 |
| BRD-K03406345 | Azacitidine | DNMT1, DNMT3A | + | 7.281690292e-25 | 10.22992388 | 7.0e-22 |
| BRD-K25737009 | CHEMBL1434137 | RARG | + | 5.457023357e-24 | 10.03300762 | 4.7e-21 |
| BRD-K78126613 | Menadione | NA | + | 7.948768006e-24 | 9.995814205 | 6.6e-21 |
| BRD-A33746814 | Avicin-G | NA | + | 2.215065301e-23 | 9.893772181 | 1.8e-20 |
| BRD-K06593056 | LE-135 | RARB | + | 3.342045763e-21 | 9.378621420 | 2.4e-18 |
| | | | | | | |
| BRD-K49865102 | PD-0325901 | MAP2K1, MAP2K2 | - | 3.662981763e-171 | 27.86381962 | 1.1e-166 |
| BRD-K79090631 | CGP 60474 | CDK1 | - | 3.560305531e-110 | 22.27062194 | 5.5e-106 |
| BRD-K57080016 | Selumetinib | MAP2K1, MAP2K2 | - | 6.995924939e-71 | 17.76170518 | 7.2e-67 |
| BRD-K13662825 | Dinaciclib | CDK1, CDK2, CDK5, CDK9 | - | 1.345923321e-53 | 15.36779274 | 8.3e-50 |
| BRD-A19500257 | 13-Hydroxy-8,14,19-trimethoxy-4 | HSP90AA1 | - | 5.786462048e-53 | 15.27299722 | 3.0e-49 |
| BRD-K87909389 | Alvocidib | CDK2, CDK4, CDK1, CDK6,… | - | 1.327234785e-52 | 15.21877700 | 5.8e-49 |
| BRD-K05104363 | CI-1040 | MAP2K1, MAP2K2 | - | 5.715129626e-48 | 14.50399301 | 1.9e-44 |
| BRD-K92571446 | WZ3105 | CLK2, CSNK1E, FLT3, ULK1 | - | 2.099340324e-47 | 14.41443383 | 6.4e-44 |
| BRD-K65182930 | NVP-AUY922 | NA | - | 1.315092399e-35 | 12.39948196 | 3.1e-32 |
| BRD-K37687095 | AZD-8330 | MAP2K1, MAP2K2 | - | 2.308711676e-35 | 12.35430272 | 5.1e-32 |
| BRD-K13390322 | AT-7519 | CDK2, CDK1, CDK9, CDK4,… | - | 7.798829060e-35 | 12.25601884 | 1.5e-31 |
| BRD-A13122391 | PG 490 | NA | - | 1.184190576e-34 | 12.22211620 | 2.1e-31 |
| BRD-A11702965 | Chromomycin-A3 | NA | - | 4.935989669e-31 | 11.52499338 | 8.0e-28 |
| BRD-K64538373 | RO4987655 | MAP2K1, MAP2K2 | - | 6.683580064e-30 | 11.29837296 | 9.8e-27 |
| BRD-A18497530 | IN1451 | MAPK3 | - | 1.497924936e-25 | 10.38195235 | 1.6e-22 |
| BRD-U00779237 | WZ-3105 | CLK2, CSNK1E, FLT3, ULK1 | - | 1.547491273e-25 | 10.37884454 | 1.6e-22 |
| BRD-K14529030 | Meisoindigo | NA | - | 1.876318929e-24 | 10.13785105 | 1.7e-21 |
| BRD-U51951544 | ZG-10 | IRAK1 | - | 1.946222495e-24 | 10.13427645 | 1.8e-21 |
| BRD-K70578146 | Dactinomycin | POLR2A | - | 3.916112230e-24 | 10.06570738 | 3.4e-21 |
| BRD-K41859756 | NVP-AUY922 | HSP90AA1, HSP90AB1 | - | 1.929794500e-22 | 9.674769896 | 1.5e-19 |
Table S1. List of the top LINCS chemical perturbagen signatures that exhibited a significant correlation with the DEP signature, as determined by the iLINCS CMap analysis. The table includes 20 chemical perturbagens with a positive correlation and an additional 20 with a negative correlation.
Table S1

## Slide 3
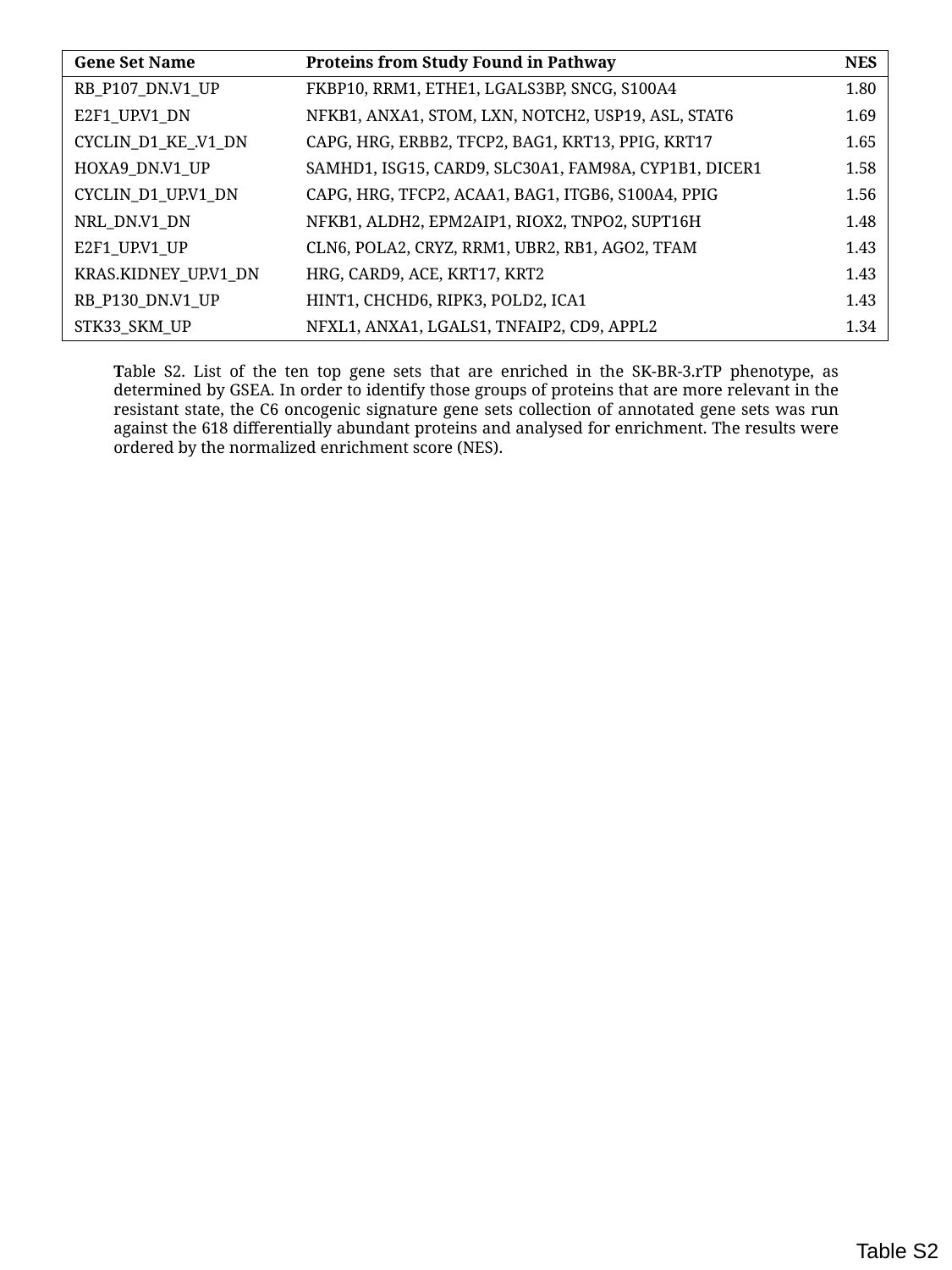

| Gene Set Name | Proteins from Study Found in Pathway | NES |
| --- | --- | --- |
| RB\_P107\_DN.V1\_UP | FKBP10, RRM1, ETHE1, LGALS3BP, SNCG, S100A4 | 1.80 |
| E2F1\_UP.V1\_DN | NFKB1, ANXA1, STOM, LXN, NOTCH2, USP19, ASL, STAT6 | 1.69 |
| CYCLIN\_D1\_KE\_.V1\_DN | CAPG, HRG, ERBB2, TFCP2, BAG1, KRT13, PPIG, KRT17 | 1.65 |
| HOXA9\_DN.V1\_UP | SAMHD1, ISG15, CARD9, SLC30A1, FAM98A, CYP1B1, DICER1 | 1.58 |
| CYCLIN\_D1\_UP.V1\_DN | CAPG, HRG, TFCP2, ACAA1, BAG1, ITGB6, S100A4, PPIG | 1.56 |
| NRL\_DN.V1\_DN | NFKB1, ALDH2, EPM2AIP1, RIOX2, TNPO2, SUPT16H | 1.48 |
| E2F1\_UP.V1\_UP | CLN6, POLA2, CRYZ, RRM1, UBR2, RB1, AGO2, TFAM | 1.43 |
| KRAS.KIDNEY\_UP.V1\_DN | HRG, CARD9, ACE, KRT17, KRT2 | 1.43 |
| RB\_P130\_DN.V1\_UP | HINT1, CHCHD6, RIPK3, POLD2, ICA1 | 1.43 |
| STK33\_SKM\_UP | NFXL1, ANXA1, LGALS1, TNFAIP2, CD9, APPL2 | 1.34 |
Table S2. List of the ten top gene sets that are enriched in the SK-BR-3.rTP phenotype, as determined by GSEA. In order to identify those groups of proteins that are more relevant in the resistant state, the C6 oncogenic signature gene sets collection of annotated gene sets was run against the 618 differentially abundant proteins and analysed for enrichment. The results were ordered by the normalized enrichment score (NES).
Table S2
